# Cardiovascular Magnetic Resonance and prognosis in cardiac amyloidosis

**DOI:** 10.1186/1532-429X-10-54

**Published:** 2008-11-25

**Authors:** Alicia M Maceira, Sanjay K Prasad, Philip N Hawkins, Michael Roughton, Dudley J Pennell

**Affiliations:** 1 Cardiac Imaging Unit – ERESA. Hospital Arnau de Vilanova, Valencia, Spain; 2 Cardiovascular Magnetic Resonance Unit, Royal Brompton Hospital, London, UK; 3 National Amyloidosis Centre, Royal Free Hospital, London, UK; 4 Medical Statistics Department, Royal Brompton Hospital, London, UK

## Abstract

**Background:**

Cardiac involvement is common in amyloidosis and associated with a variably adverse outcome. We have previously shown that cardiovascular magnetic resonance (CMR) can assess deposition of amyloid protein in the myocardial interstitium. In this study we assessed the prognostic value of late gadolinium enhancement (LGE) and gadolinium kinetics in cardiac amyloidosis in a prospective longitudinal study.

**Materials and methods:**

The pre-defined study end point was all-cause mortality. We prospectively followed a cohort of 29 patients with proven cardiac amyloidosis. All patients underwent biopsy, 2D-echocardiography and Doppler studies, ^123^I-SAP scintigraphy, serum NT pro BNP assay, and CMR with a T_1 _mapping method and late gadolinium enhancement (LGE).

**Results:**

Patients with were followed for a median of 623 days (IQ range 221, 1436), during which 17 (58%) patients died. The presence of myocardial LGE by itself was not a significant predictor of mortality. However, death was predicted by gadolinium kinetics, with the 2 minute post-gadolinium intramyocardial T1 difference between subepicardium and subendocardium predicting mortality with 85% accuracy at a threshold value of 23 ms (the lower the difference the worse the prognosis). Intramyocardial T1 gradient was a better predictor of survival than FLC response to chemotherapy (Kaplan Meier analysis P = 0.049) or diastolic function (Kaplan-Meier analysis P = 0.205).

**Conclusion:**

In cardiac amyloidosis, CMR provides unique information relating to risk of mortality based on gadolinium kinetics which reflects the severity of the cardiac amyloid burden.

## Background

Amyloidosis is an uncommon condition caused by the deposition of misfolded, insoluble aggregated protein with a characteristic β-sheet structure in tissues throughout the body [1]. Cardiac involvement is frequent in systemic amyloidosis of immunoglobulin light chain (AL) and transthyretin (TTR) types, is associated with a poor prognosis [2], and can have therapeutic implications. Accumulation of amyloid in the myocardial interstitium [3] leads to diastolic dysfunction and restrictive cardiomyopathy that progresses to overt heart failure and death [4]. Cardiac involvement is the cause of death in approximately half of patients with AL amyloidosis [5].

Cardiac amyloidosis is characterized histologically by infiltration and expansion of the interstitial space with amyloid protein, along with some associated endomyocardial fibrosis [6]. We have previously reported that cardiovascular magnetic resonance (CMR) frequently shows a characteristic pattern of global subendocardial late gadolinium enhancement (LGE) in cardiac amyloidosis that accords with the transmural histological distribution of amyloid [6]. However, there are also abnormal myocardial and blood pool gadolinium kinetics which are likely to reflect cardiac amyloid load, and therefore might relate to prognosis in these patients. We examine here the hypothesis that LGE and gadolinium kinetics might be of prognostic value in cardiac amyloidosis.

## Methods

We prospectively followed our previously reported cohort of 29 patients with proven cardiac amyloidosis from the National Amyloidosis Centre of the United Kingdom [6], who were recruited and scanned between August 2002 and April 2003. The study was approved by the local Ethical Committee and written, informed consent was obtained from all patients. The amyloidosis was of TTR type in 4 patients, and monoclonal light chain (AL) type in 25 cases. The CMR methodology and baseline characteristics of these subjects have been published previously [6]. Briefly, all patients underwent biopsy (2 cardiac, 27 non-cardiac sites), 2D-echocardiography and Doppler studies, ^123^I-SAP scintigraphy to measure extra-cardiac amyloid burden, serum NT pro BNP assay, and CMR. CMR was performed on a 1.5T scanner (Siemens Sonata, Erlangen, Germany) with acquisition of fast imaging with steady-state free precession 2 chamber, 4 chamber and contiguous short-axis breath-hold cines (7 mm slice thickness, repetition time/echo time of 3.2/1.6 ms; temporal resolution 25 ms; retrospective gating; pixel size 2.4 × 1.5 mm; flip angle 60°; acquisition time 18 heartbeats) for mass and volumes measurements. For LGE, a peripheral bolus injection (0.1 mmol/kg) of gadolinium-DTPA (Schering, Berlin, Germany) was given. A T_1 _mapping method was developed consisting of a magnetization prepared segmented FISP cine with a 30 ms increment in inversion time for each frame. This was run every 2 minutes after the gadolinium bolus starting immediately after gadolinium injection and prior to late enhancement imaging, which was only started after 10 minutes. Segmentation was 13–25 lines, with triggering every 2–3 heart beats. Subsequent to this gadolinium kinetics imaging, late gadolinium enhancement images were also acquired using a segmented inversion recovery sequence (segmentation was 13 to 25 lines, with triggering every 2 to 3 heartbeats).

Echocardiography was performed on a GE Vingmed System with the use of standard techniques, as previously described [6]. In brief, left ventricular (LV) wall thickness was measured from the M-mode at the level of the chordae. Diastolic dysfunction at the time of study entry was assessed by PW Doppler of transmitral and pulmonary venous inflow velocities. These were measured in at least 3 consecutive beats and averaged for each measurement of the following: transmitral flow – peak velocity of early (E) and late (A) filling waves; E/A ratio; E-wave deceleration time; pulmonary venous flow: peak velocity of systolic, diastolic and A reversal waves. Abnormal diastolic function was classified according to standard criteria [7,8], in 3 dysfunctional filling patterns: slow relaxation, pseudonormal, and restrictive.

For patients with AL amyloidosis, the haematological response to chemotherapy was categorized into no response, partial response or complete response, according to whether aberrant amyloidogenic serum free light chain (FLC) concentration had fallen <50%, ≥50% or completely following chemotherapy [9].

### CMR analysis

Ventricular volumes, function, and mass were analysed as previously described and compared to age and gender matched controls [10]. LGE was assessed as present (LGE+) or absent (LGE-). For analysis of the myocardial gadolinium kinetics, 2 doughnut-shaped regions of interest were drawn, incorporating the whole subendocardium (inner third of myocardium) and the whole subepicardium (outer third of myocardium) for each frame of the mapping series in a single midventricular slice; blood pool and background were also measured. An iterative computer model was used to obtain the T1 from blood, subendocardium, and subepicardium.

### Event data

The pre-defined study end point was all-cause mortality. Patients were followed-up at the National Amyloidosis Centre and events were recorded by communication with patients and/or families, their cardiologists, and general practitioners. One patient was lost to follow-up. The duration of follow-up was computed using the date of the CMR scan to the date of the end point reached. For patients who did not reach the end point, follow-up data were collected to the time of their last clinical follow-up.

### Statistical analysis

For the statistical analysis, SPSS software (version 14.0, SPSS Inc, Chicago, Illinois) was used. All non-survival quantitative variables except NT proBNP were found to conform to normality with the Kolmogorov-Smirnov test and are therefore presented as mean ± SD. The NT proBNP and survival time data are presented as median (25^th ^quartile, 75^th ^quartile). A 2-tailed Student *t*-test was used to compare continuous variables, and the χ^2 ^or Fisher's exact test was used for categorical variables. The Mann-Whitney U-test was used to compare NT proBNP between groups. A repeated-measures, mixed factorial design, 3-way ANOVA was used to analyze the change in T1 in blood, subepicardium and subendocardium with subject status (alive or dead), time after gadolinium injection, and subjects used as factors and FLC used as a covariable. Receiver operating characteristic (ROC) curves were plotted to determine the overall performance of different measurements for predicting outcome. Survival estimates and cumulative event rates were estimated using the Kaplan-Meier method. The log-rank test was used to compare the survival estimates for a number of factors. Cox proportional hazards analysis was not used because there were zero deaths in the most significant gadolinium group (intramyocardial T1 gradient at 2 minutes) which precludes this analysis, due to division by zero errors. Given the small number of patients, no multivariate analysis was done. A p value of < 0.05 was considered statistically significant.

The authors had full access to the data and take responsibility for its integrity. All authors have read and agree to the manuscript as written.

## Results

### Patient characteristics

The baseline characteristics of this cohort have been reported previously. [6] The median duration of follow-up was 623 days (221, 1436) and during this time 17 patients (58%) died. The median survival for those who died was 265 (163, 517) days. Table 1 shows the characteristics of the patients according to their status at follow-up. Among the AL amyloid patients, 3 patients in the alive group and 7 in the dead group had predominant cardiac involvement. In this group, patients with complete response to chemotherapy survived longer than those with no response (P = 0.004). Patients who died had smaller left ventricular (LV; P = 0.02) and right ventricular (RV; P = 0.045) stroke volume, and higher heart rate (P = 0.02). Patients with restrictive or pseudonormal patterns of diastolic function had a higher mortality than patients with normal or slow relaxation patterns (P = 0.04). No other significant differences were found according to LV and RV volumes and function (even when corrected for body surface area), age, gender, blood pressure and New York heart failure (NYHA) class. NT pro-BNP levels and LV mass did not show significant outcome differences. One patient was lost to follow up (AL systemic amyloid, no response to chemotherapy, no late gadolinium enhancement, gadolinium kinetics were not evaluated). Inclusion of this patient as dead or alive did not change the results of prediction of survival for the tested parameters. The results presented therefore do not include this patient.

**Table 1:** Characteristics of patients at baseline according to outcome. Table shows number or mean ± SD, except NT pro-BNP which is median (25^th ^centile, 75^th ^centile)

	Alive	Dead	P
N	11	17	
Age, years	58 ± 11	58 ± 10	0.99
Male	5	9	0.57
Type of amyloidosis			
AL	10	14	
TTR	1	3	0.67
NT pro-BNP (pMol/L) [N < 20]	462 (181, 746)	591 (456, 1176)	0.11
FLC			
No response	1	10	
Partial response	5	4	
Complete response	4	0	0.004
Systolic BP, mmHg	120 ± 10	113 ± 5	0.29
Diastolic BP, mmHg	70 ± 3	65 ± 4	0.10
Heart rate, beats/min	75 ± 13	89 ± 12	0.02
NYHA functional class			
I	6	8	
II	4	7	
III	0	2	
IV	1	0	0.67
Diastolic function pattern			
Normal	3	0	
Slow relaxation	5	7	
Pseudonormal	1	2	
Restrictive	2	8	0.04
CMR dimensions and function			
LV EDV, mL	108 ± 30	95 ± 23	0.20
LV ESV, mL	39 ± 15	41 ± 21	0.73
LV SV, mL	70 ± 21	54 ± 13	0.02
LV EF, %	63 ± 10	58 ± 12	0.19
LV mass, g	204 ± 79	201 ± 54	0.93
RV EDV, mL	111 ± 36	95 ± 24	0.18
RV ESV, mL	43 ± 20	42 ± 21	0.88
RV SV, mL	68 ± 20	54 ± 15	0.045
RVEF, %	61 ± 8	58 ± 14	0.43
RV mass, g	72 ± 21	68 ± 20	0.64
AV plane descent, mm			
LV septum	8 ± 3	6 ± 5	0.36
LV lateral wall	9 ± 4	7 ± 4	0.28
RV septum	9 ± 4	9 ± 3	0.60
RV lateral wall	15 ± 7	12 ± 5	0.25

### Late Gadolinium Enhancement and Survival

**Figure 1:**
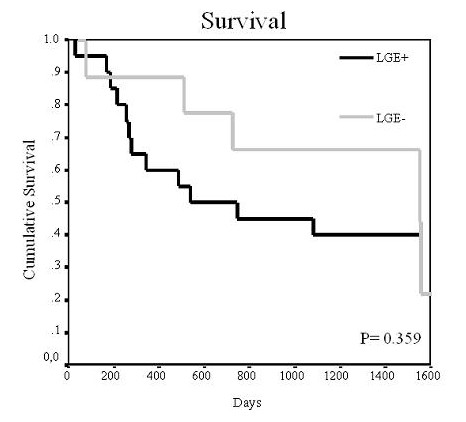
**Survival curve according to late gadolinium enhancement (LGE)**. The figure shows the Kaplan Meier curve of survival according to the presence or absence of late gadolinium enhancement (LGE). No differences in survival were seen with respect to this parameter.

There were 20 LGE+ patients. There were 5 deaths in the LGE- group and 12 in the LGE+ group (P = 0.31). Median survival was 710 (115, 1451) days for the LGE- group and 536 (252, 1415) days for the LGE+ group. Kaplan-Meier analysis showed no significant difference in survival between the 2 groups based on this parameter (P = 0.36; figure 1).

### Gadolinium Kinetics and Survival

**Figure 2:**
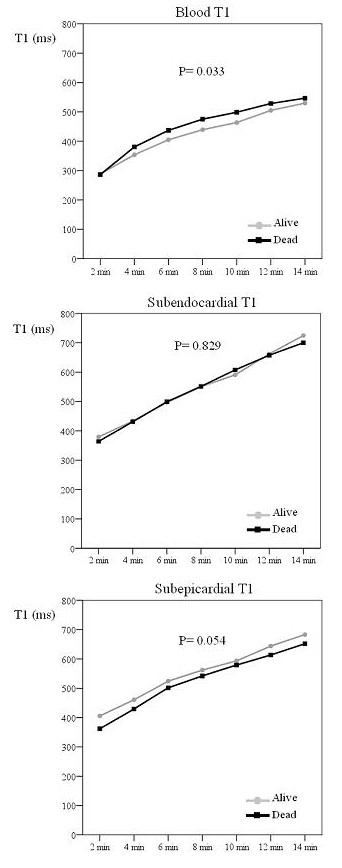
**Differences in T1 after gadolinium injection between survivors and patients who died**. The figure shows the differences in T1 of blood, subendocardium and subepicardium with time after gadolinium injection between survivors and patients who died on follow-up. Patients who died on follow up showed a higher blood T1 after gadolinium injection.

After injection, blood gadolinium clearance was faster in the patients who died, resulting in a higher blood T1 over time (P = 0.033, figure 2a), although differences were small. No significant difference was found between groups in subendocardium gadolinium clearance over time (P = 0.83. figure 2b) and the difference between groups in subepicardial gadolinium clearance showed borderline significant slower gadolinium clearance in the patients who died (P = 0.054; figure 2c).

**Figure 3:**
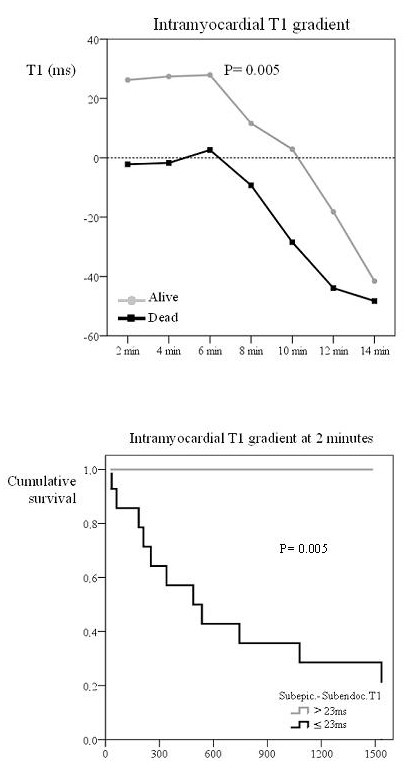
**Top: Intramyocardial T1 difference in survivors and patients who died. Bottom: Survival curve according to intramyocardial T1 difference**. The top graph shows the intramyocardial T1 difference (subepicardium – subendocardium) with time after gadolinium injection in survivors and patients who died. Survivors showed a significantly higher intramyocardial T1 difference after gadolinium injection. The bottom image shows the Kaplan Meier curve of survival according to intramyocardial T1 difference set a threshold value of 23 ms. Patients with an intramyocardial T1 difference above 23 ms had increased survival.

The intramyocardial T1 gradient (subepicardium T1 minus subendocardium T1), for which higher values indicate less gadolinium in the epicardium, showed significant differences of greater magnitude with respect to outcome. Patients who survived had a higher intramyocardial T1 gradient than the patients who died (figure 3a; P = 0.005). ROC analysis showed that an intramyocardial T1 gradient of 23 ms at 2 minutes yielded an optimal 85% accuracy for predicting survival (100% sensitivity, 66% specificity) (table 2). Kaplan-Meier curves showed significant differences in survival at the 23 ms threshold (figure 3b, P = 0.005).

**Table 2:** Diagnostic accuracy of gadolinium kinetics parameters for death. Diagnostic accuracy of gadolinium kinetics parameters for using receiver operating characteristic curve analysis (AUC = area under curve).

	AUC	P	Cut-off value	Sens (%)	Spec (%)	Accuracy (%)
Subepicardium T1 (2 min)	0.784	0.039	386	82	75	75
Intramyocardial T1 gradient (2 min)	0.833	0.012	23	100	66	85
Subepicardial -blood T1 (4 min)	0.875	0.006	80	91	88	90

**Figure 4:**
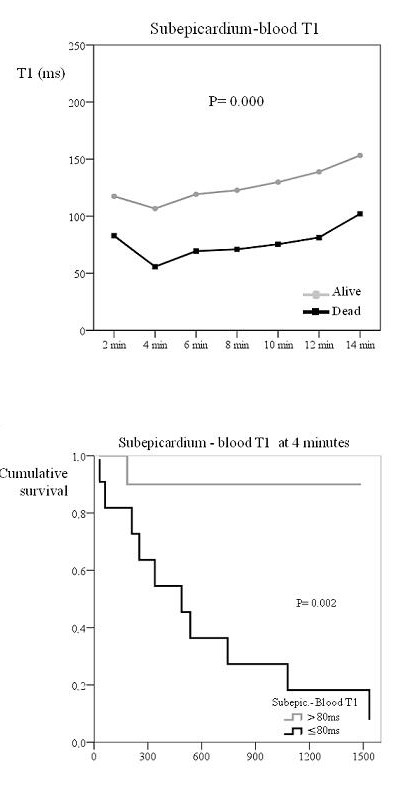
**Top: Subepicardium to blood T1 difference in survivors and patients who died. Bottom: Survival curve according to this T1 difference**. The top graph shows the subepicardium to blood T1 difference with time after gadolinium injection in survivors and patients who died. Survivors showed a significantly higher subepicardium to blood T1 difference after gadolinium injection. The bottom figure shows the Kaplan Meier curve of survival according to subepicardium to blood T1 difference set at a threshold value of 80 ms. Patients with a subepicardium to blood T1 difference above 80 ms had a better survival.

**Figure 5:**
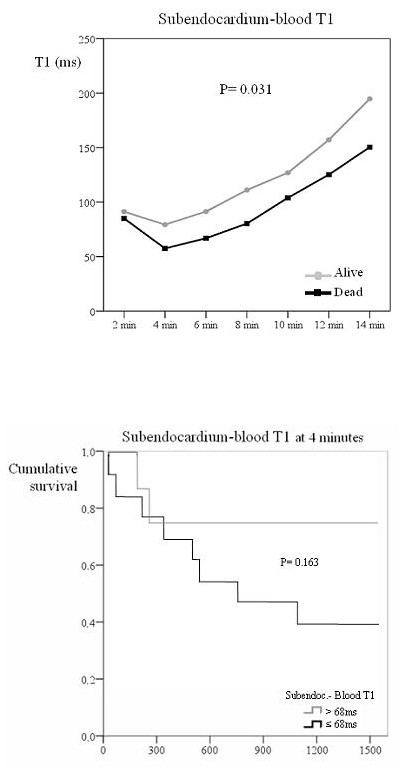
**Top: Subendocardium to blood T1 difference in survivors and those who died. Bottom: Survival curve according to this T1 difference**. The top graph shows the subendocardium to blood T1 difference with time after gadolinium injection in survivors and patients who died. Survivors showed a significantly higher subendocardium to blood T1 difference after gadolinium injection. The bottom figure shows the Kaplan Meier curve of survival according to subendocardium to blood T1 difference set a threshold value of 68 ms. No significant differences in survival were seen for this parameter.

Surviving patients also had a higher subepicardial minus blood T1 difference, again reflecting lower epicardial gadolinium uptake (figure 4a; P < 0.001). ROC curves showed that a subepicardium-blood T1 difference of 80 ms at 4 minutes yielded 90% accuracy for mortality (91% sensitivity, 88% specificity; table 2). Kaplan-Meier curves showed significant differences in mortality at the 80 ms threshold (figure 4b; P = 0.002). Surviving patients also showed a higher subendocardial-blood T1 difference (figure 5; P = 0.031) but ROC results showed that this parameter did not predict survival.

**Figure 6:**
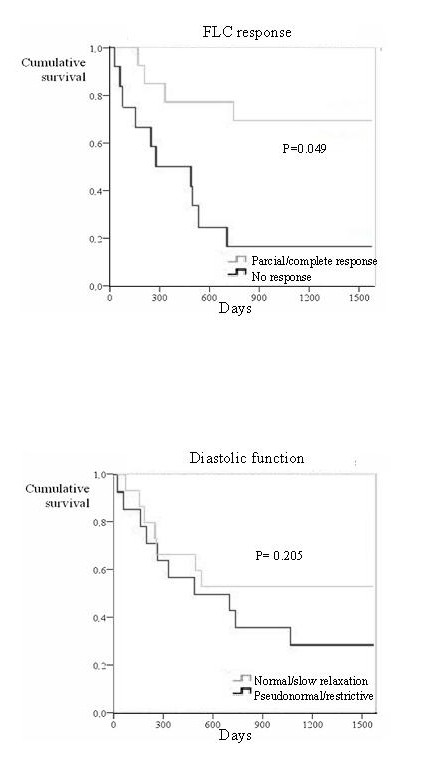
**Top: Survival curve according to FLC response. Bottom: Survival curve according to diastolic function**. The top graph shows the Kaplan Meier curve of survival according to free light chain response to chemotherapy. Patients with partial/complete response had better survival. The bottom image shows the Kaplan Meier curve of survival according to diastolic function. The discrimination of survivors from non-survivors was superior for gadolinium kinetics (figure 3).

Due to the sample size, no multivariable analysis was done and Kaplan-Meier curves were used instead to compare survival with respect to several parameters. Since both the pattern of diastolic function and FLC response to chemotherapy have been considered significant predictors of mortality in this condition, Kaplan-Meier curves were produced in order to compare differences in survival according to these two variables (figure 6). Patients with partial or complete response to chemotherapy showed a better survival than those with no response, though the degree of significance was not as good as that of gadolinium kinetics parameters (P = 0.049). Finally, no significant differences were found in survival between patients with normal/slow relaxation compared to pseudonormal/restrictive diastolic pattern.

## Discussion

Accumulation of amyloid in the myocardial interstitium results in late gadolinium enhancement (LGE), often with a predominant diffuse, global and subendocardial distribution that matches the distribution of amyloid on histology [6,11,12], although other more focal patterns have also been reported [13]. This is associated with substantial alterations in gadolinium kinetics, with faster washout of gadolinium from blood and myocardium than normal [6]. This study extends the value of these diagnostic findings, and shows for the first time that CMR may yield useful prognostic information in patients with cardiac amyloidosis. The presence alone of LGE did not correlate significantly with survival, although the sample size of this study was relatively small, but gadolinium kinetics were associated with significant survival differences according to several derived parameters. A likely explanation for the discrepancy between LGE and the kinetics with outcome is the superior discrimination by gadolinium kinetics for the severity and transmurality of the myocardial amyloid burden. This is illustrated by the results of the intramyocardial T1 difference parameter. Previous work showed that the usual pattern of amyloid protein deposition in the myocardium is predominantly subendocardial with variable transmural extension, [6] under which circumstances the subepicardial (low amyloid, low gadolinium, high T1) minus subendocardial T1 (higher amyloid, higher gadolinium, lower T1) difference would be expected to be high. With greater myocardial amyloid deposition, including greater deposition in the subepicardium, the transmural difference in amyloid diminishes, leading to a reduction in the intramural T1 gradient. This is supported by the other significantly predictive derived parameter in which myocardial T1 is compared with blood T1; the subepicardial minus blood T1 difference also predicted survival, with lower values associated with death. Usually blood contains more gadolinium (lower T1) because of a substantially greater volume of distribution, and therefore a large positive T1 gradient between subepicardium and blood exists. This gradient would be reduced if greater subepicardial amyloid (lower T1) is present. Of interest is that the subendocardial minus blood T1 difference did not show differences in survival. It therefore appears that deposition in the subepicardium may be the principal statistical driver for prediction of mortality. The explanation for this finding can be that subendocardial amyloid deposition is usual, and it is not until the burden of amyloid deposition increases that the subepicardium has substantial involvement. Thus it is possible that subepicardial involvement partially reflects the total myocardial amyloid burden. However, the direct predictive value for death of subepicardial T1 was less than the derived measures already mentioned (P = 0.039; table 2; Kaplan Meier curves not significant), and therefore the derived measures are more important.

Cardiac involvement in amyloidosis is a major risk factor for adverse outcome [14]. Previously proposed associations with poor prognosis in cardiac amyloidosis include reduced ejection fraction, low ECG voltages, increased left ventricular wall thickness on echo, and the type of amyloidosis (with worse prognosis in AL compared with TTR type) [15]. In addition, the degree of diastolic dysfunction [16] and suppression of amyloidogenic serum light chains by chemotherapy, and lower baseline values and greater reductions in NT-proBNP have been associated with improved outcome [9,17]. Our data now suggest that gadolinium kinetics may be even more predictive than these measures. The value of the CMR measurements may in part be due to the fact that cardiac amyloid burden cannot be measured satisfactorily by other techniques, and therefore CMR may offer a fundamental new window into the cardiac pathology in this disease.

Treatment options in amyloidosis have been expanding [18], and some novel pharmaceuticals have lately shown considerable promise [19,20]. Patients with a particular poor prognosis may benefit from sequential heart and autologous stem cell transplantation [21-23]. Recognition that T1 mapping in cardiac amyloidosis may be significantly more predictive of poor prognosis than the other currently used measures may be beneficial in the clinical management of AL patients by identifying those in whom early use of more intensive chemotherapy might be justified with the aim of achieving more rapid and complete remission of their underling clonal plasma cell disease. It is of interest that there is precedent for the burden of myocardial disease as assessed by CMR being predictive of outcome in hypertrophic, dilated and siderotic cardiomyopathies [24-26], with the severity of fibrosis [24,25,27] and iron loading [28].

A number of the gadolinium kinetics parameters in this study were significantly associated with mortality, but the one with greatest discriminatory value was the intra-myocardial T1 gradient after gadolinium injection, with 95% accuracy at a threshold value of 23 ms (Kaplan Meier analysis P = 0.002). Although further experience and reproduction of these results by other centres is necessary, the technique is in principle straightforward and could be implemented on most 1.5T scanners. Optimisation of the acquisition sequence and analysis software would also be valuable.

### Study limitations

Patient numbers were relatively small but cardiac amyloidosis is a rare disease. The diagnosis of cardiac amyloidosis did not routinely include endocardial biopsy, in keeping with standard clinical practice in the UK National Amyloid Centre and international consensus guidelines on diagnosis and organ involvement in amyloidosis [29]. The measurement technique for T1 includes assumptions about the relaxivity of gadolinium-DTPA being the same in blood and myocardium. Due to the small sample size, no multivariable analysis was carried out.

## Conclusion

This study shows that gadolinium kinetics assessed by T1 relaxation CMR is useful in the prognostic assessment of cardiac amyloidosis and this could have utility in assessing patients for treatments, and in their follow-up.

## Competing interests

This study was supported by CORDA and The British Heart Foundation. Research support was also received from Siemens Medical Solutions. The authors declare that they have no competing interests.

## Authors' contributions

AMM conceived the study, carried out the CMR studies, obtained and analysed the data and drafted the manuscript. SKP helped to analyse the data and draft the manuscript. PNH helped with conception and design of the study and helped to draft the manuscript. MR participated in the design of the study and the statistical analysis. DJP conceived the study, participated in its design and coordination and helped to draft the manuscript. All authors read and approved the final manuscript.
